# High OCT4A levels drive tumorigenicity and metastatic potential of medulloblastoma cells

**DOI:** 10.18632/oncotarget.15163

**Published:** 2017-02-07

**Authors:** Patrícia Benites Gonçalves da Silva, Márcia Cristina Teixeira dos Santos, Carolina Oliveira Rodini, Carolini Kaid, Márcia Cristina Leite Pereira, Gabriela Furukawa, Daniel Sanzio Gimenes da Cruz, Mauricio Barbugiani Goldfeder, Clarissa Ribeiro Reily Rocha, Carla Rosenberg, Oswaldo Keith Okamoto

**Affiliations:** ^1^ Centro de Pesquisa sobre o Genoma Humano e Células-Tronco, Departamento de Genética e Biologia Evolutiva, Instituto de Biociências, Universidade de São Paulo, São Paulo, SP, Brazil; ^2^ Departamento de Patologia, Faculdade de Medicina Veterinária e Zootecnia, Universidade de São Paulo, São Paulo, SP, Brazil; ^3^ Laboratório de Bioquímica e Biofísica, Instituto Butantan, São Paulo, SP, Brazil; ^4^ Departmento de Microbiologia, Instituto de Ciências Biomédicas, Universidade de São Paulo, São Paulo, SP, Brazil

**Keywords:** OCT4A, POU5F1, LIN28A, medulloblastoma, aggressiveness

## Abstract

Medulloblastoma is a highly aggressive pediatric brain tumor, in which sporadic expression of the pluripotency factor OCT4 has been recently correlated with poor patient survival. However the contribution of specific OCT4 isoforms to tumor aggressiveness is still poorly understood. Here, we report that medulloblastoma cells stably overexpressing the OCT4A isoform displayed enhanced clonogenic, tumorsphere generation, and invasion capabilities. Moreover, in an orthotopic metastatic model of medulloblastoma, OCT4A overexpressing cells generated more developed, aggressive and infiltrative tumors, with tumor-bearing mice attaining advanced metastatic disease and shorter survival rates. Pro-oncogenic OCT4A effects were expression-level dependent and accompanied by distinct chromosomal aberrations. OCT4A overexpression in medulloblastoma cells also induced a marked differential expression of non-coding RNAs, including poorly characterized long non-coding RNAs and small nucleolar RNAs. Altogether, our findings support the relevance of pluripotency-related factors in the aggravation of medulloblastoma traits classically associated with poor clinical outcome, and underscore the prognostic and therapeutic value of OCT4A in this challenging type of pediatric brain cancer.

## INTRODUCTION

Primary malignant tumors of the central nervous system (CNS), although rare, are challenging to treat, often associated with severe patient morbidity and high mortality rates. This is the case of some embryonal CNS tumors occurring mostly in pediatric patients [1, 2]. In young children, medulloblastoma is the predominant form of embryonal tumor, which is comprised of densely packed cells with high mitotic activity and ability to spread throughout the CNS [1]. About 40% of medulloblastoma patients already present metastasis at initial diagnosis [3], which further complicates treatment, consisting basically of surgical resection followed by craniospinal radiotherapy and chemotherapy [2].

To minimize the risk of secondary tumors, as well as considerable neurological, vascular, and endocrinal sequels of radiation therapies, children over three years of age with minimum residual disease (average risk group) receive adjuvant radiotherapy at lower doses than children with partial tumor resection and/or metastatic disease (high risk group) [2]. Following adjuvant chemotherapy, up to 60% of high risk patients still experience disease progression or succumb to the disease within 5 years [4]. As recently discussed in the CNS drug discovery and development conference [5], this significant rate of poor treatment response is partly due to the lack of effective drugs capable of reaching infiltrating tumor cells at primary and metastatic sites, and of destroying cells despite tumor heterogeneity. Another complicating factor relates to the high degree of intertumor heterogeneity observed in medulloblastoma, which is currently classified in four distinct molecular subtypes, WNT, SHH, Group 3 and Group 4 [6].

Pursuing novel therapeutic targets is also encouraged for medulloblastoma. For this particular purpose, a better knowledge of the basic biology of this important embryonal CNS tumor is crucial. Previous independent studies have shown an intriguing abnormal expression of the pluripotency-related genes *POU5F1*/*OCT4* [7], *LIN28B* [8], *SOX2* [9] and *L1TD1* [10], in medulloblastoma. In all cases, an aberrant overexpression of either gene was significantly correlated with poor survival. Intriguingly, genome-wide studies in medulloblastoma so far have not described driver mutations in such genes. A recent whole-genome methylation profiling analysis, however, did find a hypomethylation in an alternative promoter of *LIN28B* that was correlated with increased *LIN28B* expression particularly in Group 3 and Group 4 medulloblastomas [8]. These studies support a possible contribution of pluripotency-related genes in medulloblastoma physiopathology, although further functional evidence is needed.

In embryonic stem cells (ESC), LIN28 promotes *OCT4* expression indirectly, by binding its inhibitory microRNA let-7, and through direct binding of *OCT4* transcripts, thereby enhancing their translation [11]. Abnormal expression of *OCT4* has been detected in different types of aggressive cancers [12–14]. In medulloblastoma specimens, in particular, increased *OCT4* expression was shown capable of discriminating average risk patients with poorer survival typical of high risk patients [7]. Despite this prognostic value, direct evidence of *OCT4* contribution to more aggressive traits in medulloblastoma is missing.

The OCT4 transcription factor is encoded by the *POU5F1* gene located in chromosome 6. Alternative splicing of the *POU5F1* primary transcript generates five transcript variants, encoding the isoforms OCT4A, OCT4B-190, OCT4B-265, OCT4B-164 and OCT4B1 [15–17]. OCT4A is the most studied and described isoform, originally reported as a regulator of ESC pluripotency and self-renewal [18], while OCT4B and OCT4B1 functions are still uncertain. There are reports of OCT4B and OCT4B1 involvement with genotoxic stress and anti-apoptotic properties [16,19], but no clear association with stemness [20]. The identity of the OCT4 isoform predominantly involved in cancer is still elusive since no distinction has been made in most studies reporting aberrant OCT4 expression in tumors [13, 21, 22].

In light of these recent observations, when evaluating expression of *OCT4* transcript variants in medulloblastoma, we found a specific correlation between OCT4A and poor survival, as well as a potent oncogenic activity for OCT4A. These findings highlight the involvement of OCT4A in a mechanism driving aggressiveness of medulloblastoma, which could be further explored not only as a prognostic indicator, but also as a therapeutic target for a precision medicine approach in neuro-oncology.

## RESULTS

### Increased OCT4A levels enhance proliferation, tumorsphere generation capacity and invasion of medulloblastoma cells

**Figure 1:**
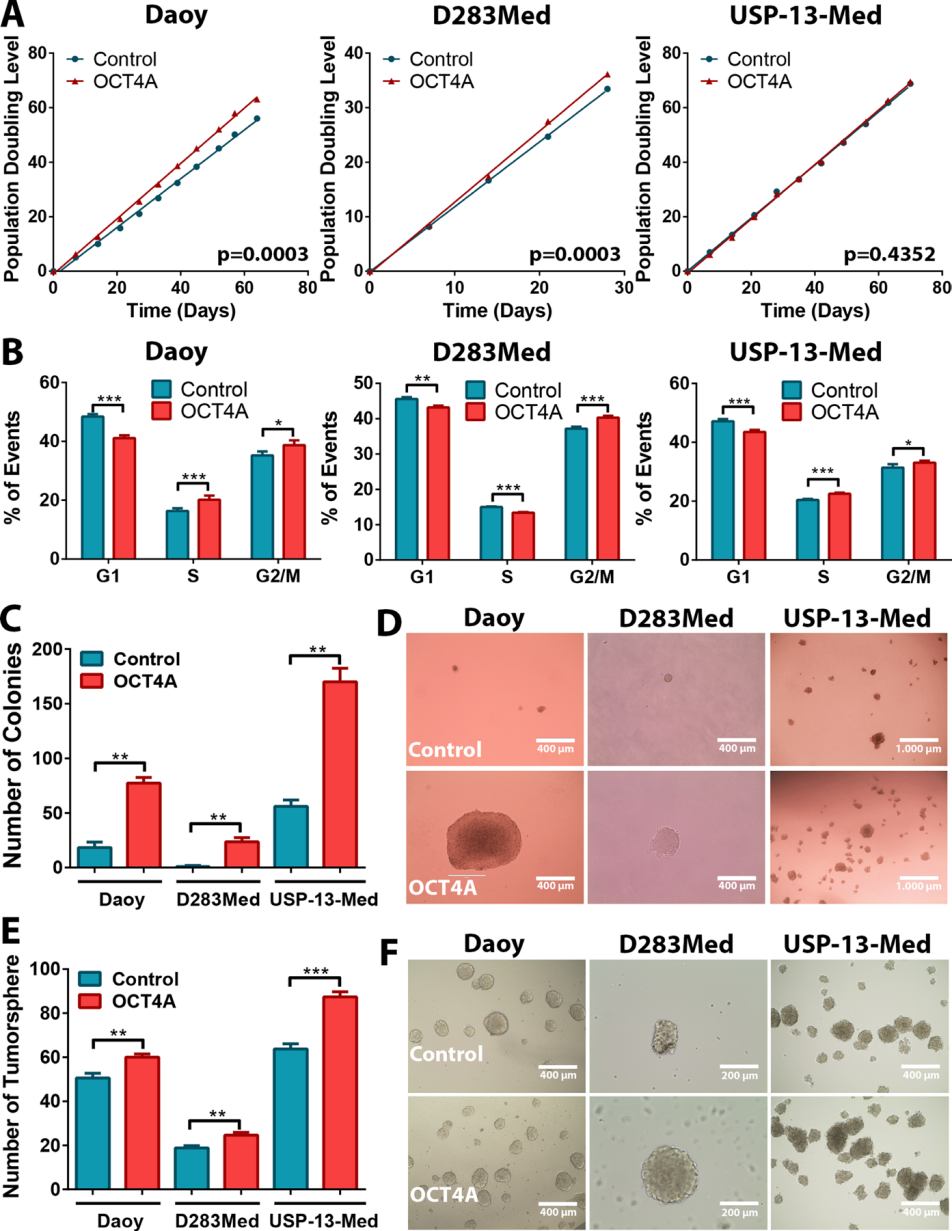
OCT4A overexpression increases medulloblastoma cell proliferation and tumorsphere generation *in vitro* (**A**) Comparative population doubling levels of parental and OCT4A-overexpressing cells after at least 30 generations. (**B**) Cell cycle analysis by flow cytometry displaying a lower proportion of cells in G1 phase and increased proportion of cells in S and/or G2/M in medulloblastoma OCT4A-overexpressing cells. The bars represent mean ± SEM of three independent experiments done in triplicate. (**C**) OCT4A overexpression and enhanced medulloblastoma cell clonogenicity. Bar graph indicates the total amount of cell colonies generated under anchorage-independent growth conditions. Only colonies larger than 50 μm were counted. The bars represent mean ± SEM of two independent experiments done in triplicate. (**D**) General aspect of Daoy, D283Med and USP-13-Med colonies. Colonies of OCT4A-overexpressing cells were increased in size. (**E**) Medulloblastoma OCT4A-overexpressing cells also displayed significantly enhanced tumorsphere generation capability. The bars represent mean ± SEM of three independent experiments done in triplicate. (**F**) Representative images of medulloblastoma tumorspheres. *p < 0.05, **p < 0.01, ***p < 0.001.

Expression of *LIN28A* and *POU5F1* has been correlated in medulloblastoma [7]. Here, a more detailed analysis revealed that, from all alternative *POU5F1* transcripts investigated, only OCT4A transcript levels significantly correlated with *LIN28A* expression in clinical medulloblastoma specimens (Supplementary Figure 1). Given a previous correlation of OCT4A expression with poor patient survival [7], we next evaluated whether OCT4A would directly affect aggressive traits of medulloblastoma cells. Stable OCT4A-overpressing medulloblastoma cell lines were generated and characterized to confirm specific enhancement of OCT4A [23]. Western blot assays indicated that the OCT4A overexpression in tumor cells yielded OCT4 protein levels that were lower than the levels found in normal human ESC, thus, within physiological levels (Supplementary Figure 2). Population doubling level (PDL) assays carried out for at least 30 generations revealed a significant decrease in population doubling time of Daoy and D283Med cells upon OCT4A overexpression (Figure 1A). Accordingly, a significant shift in cell cycle towards increased proportion of cells in S and G2/M phases and decreased proportion of cells in G1 was observed for all medulloblastoma cell lines stably overexpressing OCT4A (Figure 1B). These results indicate that OCT4A increase proliferation of medulloblastoma cells.

**Figure 2:**
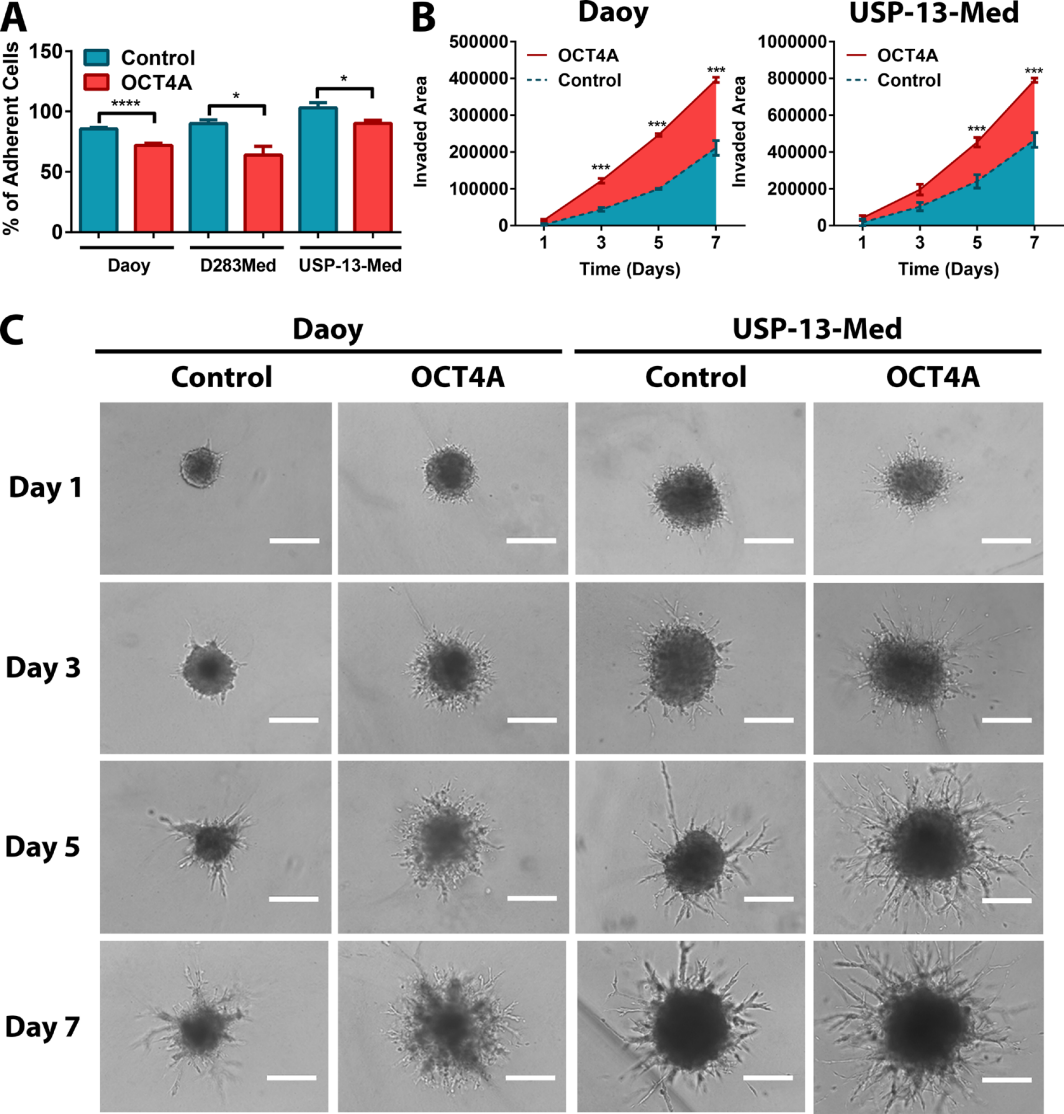
OCT4A significantly potentiates medulloblastoma cell motility (**A**) Tumor cells overexpressing OCT4A exhibited significantly reduced adhesion properties. The bars represent mean ± SEM of three independent experiments done in triplicate. (**B**) 3D cell invasion assay with OCT4A-overexpressing cells displaying a significantly enhanced invasive behavior. Data are displayed as area of invading protrusions emerging from the initial tumor spheroid area. Blue: Control (parental cells); Red: OCT4A-overexpressing cells. The curves represent mean ± SEM of three independent experiments done in quadruplicate. (**C**) Representative images of tumor spheroid cells invading the hydrogel matrix at days 1, 3, 5 and 7. *Bar size: 400 μm*. *p < 0.05, ***p < 0.001, ****p < 0.0001.

**Figure 3:**
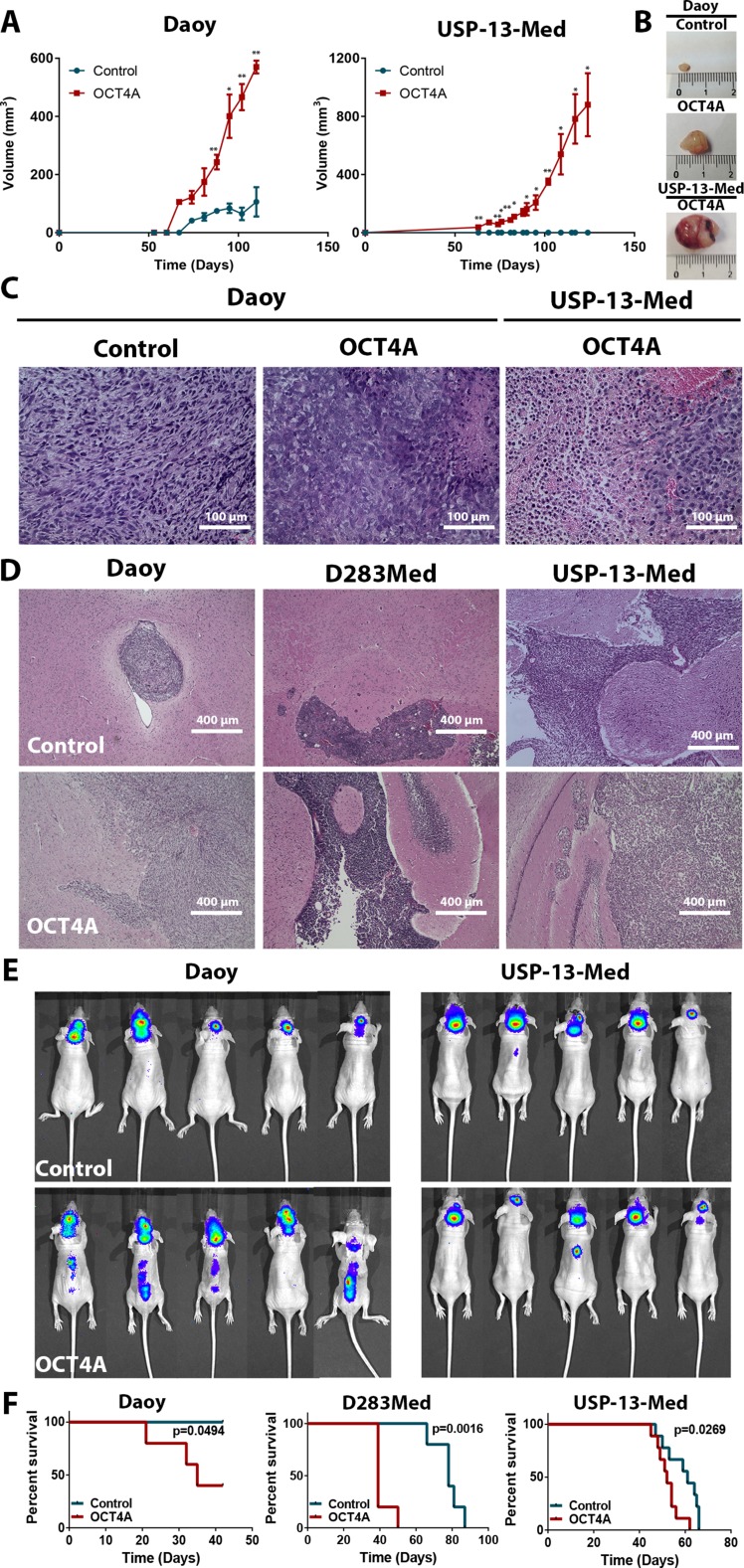
OCT4A overexpression significantly enhances tumorigenicity of medulloblastoma cells (**A**) Kinetics of tumor growth after subcutaneous inoculation of medulloblastoma cells in BALB/c nude mice. OCT4A-overexpressing cells generated more developed tumors at a faster pace than corresponding control cells. *p < 0.05, **p < 0.01. (**B**) Representative images of subcutaneous tumors resected after 90 or 120 days post-injection of Daoy or USP-13-Med cells, respectively. Control USP-13-Med cells and D283Med cells did not generate palpable subcutaneous tumors under the experimental conditions tested. (**C**) Histological analysis showing typical aggressive features, including extensive necrotic and hemorrhagic areas, in subcutaneous tumors from OCT4A-overexpressing cells. (**D**) Representative images of histological brain sections from BALB/c nude mice bearing orthotopically implanted medulloblastoma cells. Local tumor cell invasion was typically observed in brain tumors generated from OCT4A-overexpressing cells. (**E**) Bioluminescence-based detection of medulloblastoma cells orthotopically injected in BALB/c nude mice. Images were taken three or four weeks after the intracerebroventricular injection of Daoy or USP-13-Med cells, respectively. Multiple small tumor foci, including spreading to the spinal cord were more frequent in mice injected with OCT4A-overexpressing cells. (**F**) Kaplan-Meier curves showing shorter overall survival rates for BALB/c nude mice bearing orthotopically implanted OCT4A-overexpressing cells.

Similar pro-oncogenic effects of OCT4A were observed when cells were cultured as tumor spheroids in 3D assay platforms. OCT4A overexpression significantly enhanced anchorage-independent cell growth (Figure 1C). Not only was the amount of tumor cell colonies significantly increased but also the overall size of these colonies (Figure 1D). Generation of tumorspheres enriched in stem-like cells was also significantly enhanced by OCT4A overexpression (Figure 1E–1F). Eventual increment in tumorsphere size was found to be a consequence of sphere expansion and events of sphere fusion, the latter enhanced by the frequency of such tumorspheres in culture (Supplementary Video 1). Finally, OCT4A overexpression significantly affected adhesion (Figure 2A) and 3D invasion capacity of medulloblastoma cells (Figure 2B–2C).

Noteworthy, further assays with clonal cell lines expressing increasing stable levels of OCT4A (basal, moderate and high) revealed that these pro-oncogenic effects are expression-level dependent (Supplementary Figure 3). These results are in agreement with previous clinical evidence, given that, not only frequency, but also increased OCT4 expression, have been correlated with poor survival of medulloblastoma patients [7].

### OCT4A contributes to the development of aggressive tumors *in vivo*

OCT4A overexpression significantly aggravated tumor formation and progression, as indicated by both ectopic and orthotopic models of medulloblastoma. When subcutaneously inoculated in Balb/C Nude mice, medulloblastoma cells overexpressing OCT4A generated tumors faster than respective control cells (Figure 3A–3B). While Daoy control cell-derived tumors displayed a more homogenous histological phenotype, tumors generated from Daoy OCT4A-overexpressing cells were larger and presented histologic features typical of aggressive tumors, including cells with high nucleus-to-cytoplasm ratio, presence of necrotic areas and inflammatory infiltration (Figure 3C). More striking tumorigenic effects of OCT4A were observed in experiments with the USP-13-Med cell line. USP-13-Med cells overexpressing OCT4A, but not respective control cells, were capable of generating subcutaneous tumors characterized by being highly vascularized and necrotic (Figure 3A–3B). Histological analysis of these USP-13-Med-derived tumors confirmed presence of intratumoral hemorrhagic areas with extensive inflammatory infiltration (Figure 3C). Under the same experimental conditions, experiments with D283Med cells were not conclusive since only one out of five animals injected with cells developed subcutaneous tumors in both experimental groups.

Pro-tumorigenic effects of OCT4A were also evident in an orthotopic metastatic model of medulloblastoma, in which OCT4A overexpression was found to significantly increase aggressiveness of tumors derived from all cell lines tested. Clinical symptoms were more frequent or developed earlier in animals injected with OCT4A-overexpressing cells. Similarly, except for Daoy-derived tumors, an increased metastatic spread was observed for OCT4A-overexpressing cells in histological analysis of brain sections (Table 1). Furthermore, tumors derived from OCT4A-overexpressing cells tended to be more developed, more enriched in necrotic areas, and with increased intraparenchymal invasion capability, when compared with tumors generated from control cells (Figure 3D and Supplementary Figure 4A). In the case of D283Med cells, tumor spreading to the spinal cord was found in all animals injected with OCT4A-overexpressing cells, but only in 50% of animals injected with control cells (Table 1 and Supplementary Figure 4B). These results were confirmed by longitudinal *in vivo* imaging studies of medulloblastoma-bearing mice, which showed a bimodal pattern of metastatic tumor foci in the brain and/or distant metastatic foci in the spinal cord occurring predominantly in animals injected with OCT4A-overexpressing cells (Figure 3E). Mice injected with OCT4A-overexpressing cells also displayed shorter survival (Figure 3F). Again, as observed for clonogenic activity and neurosphere generation capability, the positive effects of OCT4A on tumor development were expression-level dependent (Supplementary Figure 5).

**Table 1:** Clinical and pathological parameters of BALB/c nude mice bearing orthotopically implanted human medulloblastoma cells

	Daoy		D283Med		USP-13-Med	
	Control	OCT4A	Control	OCT4A	Control	OCT4A
**No of animals with clinical symptons**	3/5	5/5	4/4	3/3	7/8	8/10
**Days to the onset of clinical symptoms**	40.67 ± 2.31	24.40 ± 9.90*	73.8 ± 6.46	37.60 ± 5.32*	49.43 ± 9.38	50.00 ± 9.15
**No of animals with M2**	2/3	2/3	2/4	0/3	1/4	3/5
**No of animals with M3**	0/5	4/5	2/4	3/3	1/4	1/5

^*^M2 and M3 correspond to progressive stages of medulloblastoma metastasis according to Chang's staging system.

### Chromosomal aberrations and non-coding RNA expression changes associated with OCT4A overexpression

**Figure 4:**
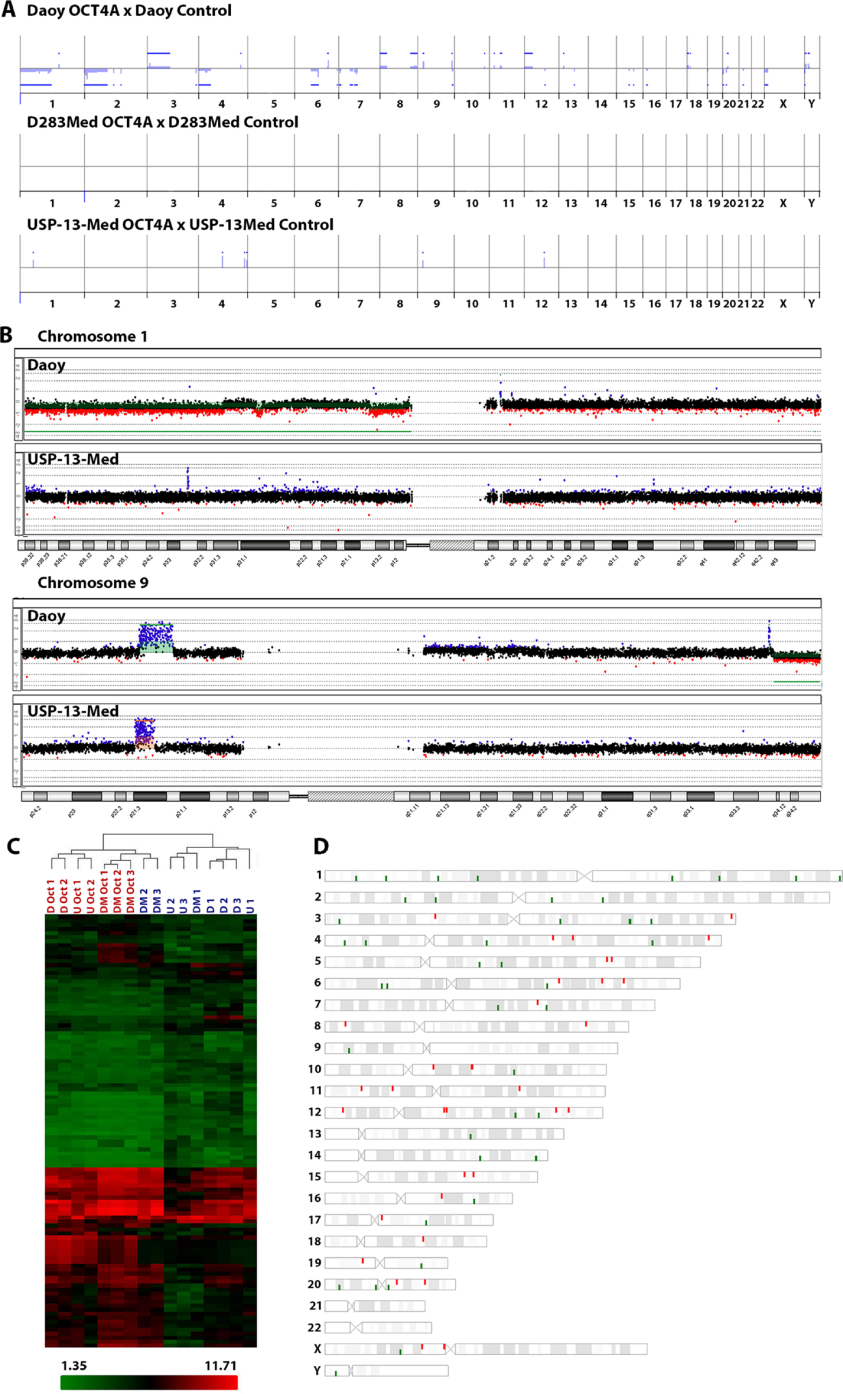
Molecular alterations induced by OCT4A overexpression in medulloblastoma cells (**A**) Differences in copy number profiles of the OCT4A overexpressed cells compared to their parental tumor cells. All chromosomes from 1p (left) to 22q, X and Y are represented. The blue bars above and below the middle lines (zero or no difference) represent more and less copy numbers, respectively. (**B**) Differences in copy number profiles of chromosomes 1 and 9 for OCT4A overexpressed medulloblastoma cell lines. Daoy, differences are highlighted in Green and USP-13-Med in Orange. (**C**) Cluster analysis of commonly differentiated expressed genes due to OCT4A overexpression. D: Daoy Control; DM: D283Med Control; U: USP-13-Med Control; D Oct: Daoy overexpressing OCT4A; DM Oct: D283Med overexpressing OCT4A; U Oct: USP-13-Med overexpressing OCT4A. Data are presented as average normalized signal in Log_2_. Genes were functionally classified according to Ensembl definition. (**D**) Chromosomal location of commonly differentiated expressed genes after OCT4A overexpression in Daoy, D283Med and USP-13-Med cells.

Since chromosomal aberrations are frequently detected in pluripotent stem cells [24], aCGH analyzes comparing tumor cells with or without OCT4A overexpression were then performed to verify whether enforced OCT4A expression would enhance genomic instability in cancer cells. Among the cell lines studied, the highest and lowest levels of OCT4A were detected in Daoy and D283Med cells, respectively. Interestingly, the highest amount of extra chromosomal aberrations associated with OCT4A overexpression was also detected in Daoy cells (39 losses and 23 gains detected). Comparatively, only few additional chromosomal aberrations due to OCT4A overexpression was detected in USP-13-Med (6 gains), while no extra aberrations were observed in D283Med cells. These copy number aberrations are presented in Figure 4A and detailed in Supplementary Data 1. Two chromosome regions were commonly found altered in Daoy and USP-13-Med cell lines after OCT4A overexpression, mapped at 1p32.3 and 9p21.3 (Figure 4B). From these, only 9p21.3 displayed the same type of aberration (amplification) in both cell lines, while a large segment in 1p was deleted in Daoy and a focal high level amplifications comprising only 2 genes (FAF1, CDKN2C) were observed in USP-13-Med after OCT4A overexpression. The genes located in the affected regions are listed in Supplementary Table 1.

Large scale changes in gene expression also occurred in medulloblastoma cells as a result of OCT4A overexpression. Considering the gene expression profiles of three independent clones of each parental and respective OCT4A-overexpressing cells, a total of 141 genes were found differentially expressed, common to all cell lines evaluated (Supplementary Table 2). Interestingly, only 43 of them encode proteins, including *POU5F1B*, a transcribed pseudogene of *POU5F1* that has been reported to encode a functional protein with transcription factor activity similar to OCT4 [25]. The remaining 98 genes (69.5%) were related to non-coding RNAs. Two other transcribed pseudogenes of *POU5F1, POU5F1P3* and *POU5F1P4*, for which proteins have not been described, were also differentially expressed (Figure 4C). Functional annotation analysis revealed enrichment of genes involved in cellular movement, cell death and survival, cellular development and cell cycle, which are all important mechanisms in tumor initiation and progression. The chromosomal location of these genes and pseudogenes affected by OCT4A overexpression is depicted in Figure 4D. Further analysis integrating chromosomal aberrations and global gene expression profiling data indicated that only a minor portion of differentially expressed genes/pseudogenes were located in regions with chromosomal copy number changes associated with OCT4A overexpression (Supplementary Tables 3, 4). Interestingly, expression of *Nanog* and *Sox2*, as well as SSEA-4, another typical pluripotency marker, was not significantly changed by OCT4A overexpression in tumor cells (Supplementary Figure 6).

## DISCUSSION

In ESC, fine-tune regulation of OCT4 levels is required for pluripotency maintenance or induction of cell differentiation [26]. OCT4 translation is favored by the RNA-binding protein LIN28, which interacts with RNA helicase A and OCT4 transcripts forming more efficient translation complexes [11]. Such translational regulation mechanism was found preserved in medulloblastoma cells. Both OCT4 and LIN28 have been implicated in malignancies [7, 27, 28], but only in a few cases concomitant expression of *OCT4* and *LIN28* have been simultaneously analyzed and correlated with prognosis [29, 30]. In our study, a significant positive correlation between expression of *LIN28A* and *OCT4A* was found in medulloblastoma samples. Interestingly, no such correlation with *LIN28A* was found with the other alternative transcripts encoding either OCT4B or OCT4B1 isoforms.

Although high *OCT4* expression has been associated with poor prognosis in different cancers [12–14], little is known about its function in tumorigenesis, particularly in brain tumors. In gliomas, few studies have found a correlation of *OCT4* expression with higher tumor grade [31], although association with patient survival was not found [32]. Studies about OCT4 isoforms in tumor development and cancer prognosis are even scarcer. Different functions have been proposed for each of these isoforms, including involvement in genotoxic stress [17], apoptosis [19, 20], and self-renewal/pluripotency [15]. Lack of isoform discrimination in most studies concerning *OCT4* expression in cancer pathogenesis [21] requires a more careful interpretation and may explain some discrepancies in the literature [33, 34]. For instance, some studies reporting OCT4 as a transcription factor involved in stem cell fate determination and cancer stemness display a predominant cytoplasmic OCT4 immunostaining [13, 22]. Cytoplasmic localization has been proposed for the OCT4B isoform, which is implicated in genotoxic stress response, whereas the OCT4A isoform involved in pluripotency is nuclear.

In a previous study, we postulated OCT4 expression as a predictor of poor clinical outcome of medulloblastoma patients, since it could discriminate patients that, despite being clinically stratified as average-risk, displayed a poor overall survival typical of high-risk patients [7]. Here, further functional studies highlight the specific contribution of the OCT4A isoform to medulloblastoma cell traits classically associated with poor prognosis, namely cell proliferation, 3D invasion behavior, clonogenicity in soft-agar, tumorsphere generation capability, and tumorigenicity. Interestingly, most of these effects were found positively correlated with the level of OCT4A expression. These findings are in agreement with the previous correlation found between increased OCT4 expression and poorer survival of medulloblastoma patients [7]. *Ex vivo* tumorsphere generation capability, in particular, is a stem cell-like property of tumor cells that has been associated with high tumorigenicity [35] and poor clinical outcome of patients with embryonal brain tumors [36], as well as other malignant tumors of the CNS [37, 38].

From the clinical point of view, of particular importance was the fact that OCT4A overexpression significantly enhanced medulloblastoma cell mobility properties *in vitro*, as well as metastatic spread in the neuroaxis, including capacity of tumor colonization in the spinal cord. Presence of metastasis at diagnosis is a classic indicator of poor prognosis in medulloblastoma patients. Tumor spread is mostly detected within the neuroaxis, where initial presence of tumor cells in the liquor progressively form nodular metastasis in supratentorial regions and/or in the spinal cord, the latter site representing the highest stage of tumor spreading (M3). Extraneuroaxial metastases (M4) are rare and associated with more advanced disease without proper treatment [4].

Notably, some non-coding RNAs differentially expressed as a consequence of OCT4A overexpression in medulloblastoma cells have been associated with poor prognosis. Elevated levels of *SNORD14C* have been suggested as prognostic marker of high risk squamous cell carcinomas of the larynx [39], while snoRA42 has been implicated in self-renewal and tumorigenesis of lung tumor initiating cells [40]. Interestingly, increased expression levels of *GNL3* (also known as *NS*, encoding the protein Nucleostemin) were also observed after OCT4A overexpression. Nucleostemin is a nucleolar protein implicated in self-renewal of ESC [41, 42] and neural stem cells [43]. Ectopic overexpression of *NS* induces dedifferentiation of somatic cells and reprogramming to a pluripotent stem cell state [41]. In cancer, increased Nucleostemin levels were correlated with high grade gliomas [44] and with cancer cells displaying enhanced tumor initiating capability [45–47]. Moreover, alterations in cell proliferation, invasion and metastasis have been observed after manipulation of *NS* expression in different cancer cells [44–46, 48, 49].

In medulloblastoma cells, OCT4A overexpression also induced chromosomal copy number aberrations, which varied in frequency and type according to the cell line. Two common mutated regions, 1p32.3 and 9p23.1, were detected in Daoy and USP-13-Med cells overexpressing OCT4A. Interestingly, trisomies of chromosomes 1 and 9 have been reported in human somatic cells after reprogramming to a pluripotent state [50]. These findings are in agreement with the notion that aberrant expression of typical pluripotency genes have oncogenic effects and may contribute to an increasing acquisition of heterogeneous chromosomal aberrations stemming from stem-like cancer cells [51]. However, the vast majority of differentially expressed genes detected in highly aggressive OCT4A overexpressing medulloblastoma cells were not located in chromosomal regions further disturbed in those cells. Altogether, these results indicate that increased OCT4A levels significantly enhance medulloblastoma cell aggressiveness through independent underlying mechanisms involving differential expression of non-coding RNAs and heterogeneous genomic aberrations. Our findings support the relevance of pluripotency-related factors in the aggravation of medulloblastoma development, as well as the prognostic and therapeutic value of OCT4A in this challenging type of pediatric brain cancer.

## MATERIALS AND METHODS

### Cell culture and patient sample

Medulloblastoma cell lines Daoy and D283Med were purchased directly from ATCC (Manassas, VA, USA) and cultured as previously described [23]. USP-13-Med is a patient-derived cell line established in our group and its cultivation method and characterization were described previously [52]. The H9 cell line of human ESC (hESC) was kindly provided by Laboratório Nacional de Células-tronco Embrionárias (LANCE, Rio de Janeiro, RJ, Brazil) and cultured under standard conditions, as previously described [62]. Cultures were maintained in a humidified 5% CO_2_ atmosphere at 37°C and subcultured when 80% of confluence was reached.

### Quantitative real time PCR

RNA was extracted from samples using RNeasy^®^ Mini Kit (Qiagen) and genomic DNA was removed by treating with DNAse I following manufacturer recommendations. RNA samples were reverse transcribed into cDNA using SuperScript^®^ II Reverse Transcriptase System (Life technologies) according to manufacturer's instructions. Primers were designed to specifically amplify OCT4A, OCT4B, and OCT4B1. Their specificity was determined by melting curve analysis, amplicons electrophoresis and sequencing (Supplementary Figure 7). Reactions were done in triplicate using Power SYBR Green Master Mix (Life technologies) on Applied Biosystem 7500 Real-Time PCR System. Quantitative analyses were performed using relative quantification curve in which human embryonic stem cell (H9) was used as positive control. Primers sequences are listed in Supplementary Table 5. Primer sequences for *Nanog* and *Sox2* are described in [53].

### Western blot

The protein extracts were submitted to western blotting standard protocol. Proteins were transferred to poly(vinylidene) difluoride (PVDF) membranes (GE Healthcare). The antibodies used were mouse anti-OCT4A antibody (Santa Cruz Biotechnologies, Dallas, TX, USA), anti-mouse IgG, HRP-linked antibody (Cell Signaling, Danvers, MA, USA) and anti-beta Actin HRP-linked antibody (Abcam). The immunoblots were developed using the ImmobilonTM Western Chemiluminescent HRP Substrate (Millipore, Billerica, MA, USA).

### *OCT4A* overexpression

All medulloblastoma cell lines underwent stable *OCT4A* overexpression by retroviral transduction (Addgene, Cambridge, MA). Briefly, HEK 293-T cells were transfected with 10% Polyethylenimine (PEI), 700 ng/mL CMV-GP plasmid, 350 ng/mL VSVG plasmid and 1 μg/mL OCT4A plasmid. Viral supernatant was collected after 48 hours, concentrated by ultracentrifugation (60.000 × g for 2 hours) and suspended in residual media overnight. Medulloblastoma cells were exposed to retroviral particles for 24 hours and expanded in culture for further analysis. *OCT4A* overexpression was confirmed at the transcriptional and protein levels by quantitative real time PCR and western blot, respectively.

### Population doubling level

To evaluate the proliferative profile of medulloblastoma cells after OCT4A overexpression, a cumulative population doubling level (PDL) was performed as previously described [52]. The PDL of parental Daoy and USP13-Med cells, under the same experimental conditions, are known [52].

### Cell cycle analysis

The cell cycle was analyzed using Cell Cycle Reagent Kit (Millipore) following manufacturer's procedure. The cell cycle was previously synchronized by serum starvation. Analyses were performed using Guava EasyCyte 5HT™ Flow Cytometer and GuavaSoft 2.1 software (Millipore).

### Soft agar colony formation assay

Single cells at low densitiy (52.63 cells per cm^2^) were seeded over a coating of 0.6% agarose solution containing supplemented media at normal conditions. The cells were allowed to set up for 10 minutes at room temperature and then covered by a 0.3% agarose solution. Plates were incubated at 37°C with 5% CO_2_ humidified atmosphere and media were replaced every 3-4 days. After 15 days, colonies over 50 μm were counted.

### Tumorsphere formation assay

Cells were seeded into a 96-well ultra-low attachment plate (Corning, Corning, NY, USA) in DMEM/F12 supplemented with B-27, N-2, 20 ng/mL of EGF and 20 ng/mL of bFGF. The tumorspheres were counted after 7 days of incubation at 37°C with 5% CO_2_ humidified atmosphere. Time-lapse of tumorsphere formation was conducted every 30 minutes for 4 days.

### Adhesion assay

Cells at a density of 8×10^3^/mL were allowed to attach into a 24 well-plate. D283Med were plated in 96 well-plate previously coated with Poly-L-ornithine 0.01% (Sigma-Aldrich, Saint-Louis, MO, USA). Non-attached cells were discarded after 45 minutes and residual cells were incubated with MTT solution (166.67 μg/mL; Sigma-Aldrich) for 4 hours at 37°C. As control of total adhesion, cells were not discarded after 45 minutes. The supernatant was discarded and formazan crystals were dissolved with DMSO. The optical density was measured at 560 nm.

### 3D spheroid invasion assay

Spheroid invasion capacity was evaluated using Cultrex^®^ 3D Spheroid Cell Invasion Assay (Trevigen, Gaithersburg, MD, USA) following the manufacturer's recommendation. Invasion area of the spheroids was measured as recommended by the manufacturer on days 0, 1, 3, 5 and 7 after invasion matrix addition. The D283Med cell line was not included in this assay since these cells fail to form spheroids under such experimental condition [23].

### Subcutaneously tumor xenograft assay

Female Balb/C Nude mice were inoculated subcutaneously in the right flank with 2×10^6^ cells (*n* = 5 per experimental group). Tumor growth were observed and measured weekly. Paraffin sections were subjected to standard H.E. procedures and analyzed under a light microscope.

### *In vivo* metastasis assay

The model used was adapted from Studebaker et al. [54]. Briefly, cells (1×10^6^) contained in 4-6 μL were stereotaxically injected in the right lateral ventricle of female Balb/C Nude mice in a ratio of 1 μL/min (*n* = 10 per experimental group for DAOY and USP-13-Med; *n* = 5 per experimental group for D283Med). The coordinates used were 1 mm to the right and 0.5 mm posterior of the bregma and 2.2 mm of depth. Animals were euthanized after 50 days of inoculation or after the development of neurological deficits and/or excessive body weight loss. The study was approved by the ethics committee for animal research of the University of São Paulo (CEUA protocol no. 132/2011). The brain and medulla were collected to standard H.E. procedure. A total of 10 sections were obtained randomly from each brain and medulla to analyze the presence of metastasis. Metastasis classification was based on M-Stage proposed by Chang [4].

Tumor development was also assessed by *in vivo* imaging using a bioluminescence-based method with the IVIS Imaging System (PerkinElmer, Waltham, MA, USA). Medulloblastoma cells were engineered to constitutively express firefly luciferase using pLV/Luc lentiviral vector, described by Rocha et al. [55] and orthotopically injected in Balb/C Nude mice as described above. Bioluminescence images were taken every week after intraperitoneal injection of 1.5 mg D-luciferin (Promega, Madison, WI, USA) in PBS. Tumor burden was calculated by the Living Image 3.1.0 software (PerkinElmer).

### Array-CGH

DNA was extracted using standard cholorophorm/phenol protocol. Array-based Comparative Genomic Hibridization (aCGH) was performed for detecting copy number alterations using 60K whole-genome platform (Agilent Technologies, Santa Clara, CA, USA). All procedures were carried out following the manufacturer's recommendation. Microarray scanned images were processed using the Feature Extraction Software and copy number aberrations (CNAs) were called using the statistical algorithm ADM-2 (sensitivity threshold: 6.7) in Genomic Workbench 6.9 6 Software (both from Agilent Technologies). Chromosome deletions and duplications were considered when log2 ratio of Cy3/Cy5 intensities were detected < −0.3 and > 0.3, respectively. Hybridizations were carried out using the OCT4A overexpressing cell lines as test samples and their corresponding tumor parental cells as reference sample.

### Global gene expression analysis

Total RNA of 2-3 clones of each cell line (Control and OCT4A overexpression) was extracted with the RNeasy kit (Qiagen), following the manufacturer's protocol. Gene expression profiling were carried out independently for each sample using Affymetrix GeneChip^®^ Human Gene 2.0 ST whole-transcript arrays (Affymetrix, Santa Clara, CA, USA). The quality control and normalization of data were processed by Affymetrix^®^ Expression Console Software (Affymetrix). Differentially expressed genes were identified with the One-Way ANOVA, with a *p-value* cutoff of 0.05, using Transcriptome Analysis Console v3.0 (Affymetrix). Functional Annotation was conducted on Ingenuity Pathway Analysis (Qiagen). Raw data is available at GEO with accession number: GSE77947.

### Statistical analysis

Unpaired Student's *t* test and Spearman's rank correlation coefficient were performed with Graph Pad Prism 6 (GraphPad Software Inc., La Jolla, CA, USA). Statistical significance was established at *p <* 0.05 level in all analyzes. Experiments were conducted in triplicate and three independent experiments were carried out. Data are presented as the mean ± SEM.

## SUPPLEMENTARY MATERIALS TABLES AND FIGURES








