# Supplementary material for: High OCT4A levels drive tumorigenicity and metastatic potential of medulloblastoma cells
Source: Oncotarget. 2017 Feb 7;8(12):19192–204. doi: 10.18632/oncotarget.15163 (PMC5386677; doi:10.18632/oncotarget.15163)
Supplement: Supplementary file 1 [file oncotarget-08-19192-s001.pdf]

High OCT4A levels drive tumorigenicity and metastatic potential of medulloblastoma cells

Supplementary Materials

LIN28A Overexpression

LIN28A was transiently overexpressed in Daoy and D283Med after cell transfection with a plasmid containing *LIN28A* fused to a GFP tag, using lipofectamine LTX (Life Technologies) and following the manufacturer's instructions. The GFP empty plasmid was used as a negative control. Cell extracts expressing either GFP or GFP-LIN28A were generated in buffer containing 50 mM Tris/HCl pH 8.0, 150 mM sodium chloride, 0.2% v/v Triton X-100, 1mM PMSF.

Co-immunoprecipitation of RNAs

Total cellular extracts were prepared from DAOY cells and added to IgG-Sepharose beads (GE Healthcare, Little Chalfont, UK) or IgG Sepharose beads conjugated with anti-LIN28A antibody (ABcam, Cambridge, UK). Immunoprecipitation was performed at 4°C for 4 h. IgG-Sepharose beads were washed with buffer containing 10 mM Tris-Cl pH 7.5, 5 mM magnesium chloride, 0.1% Nonidet P-40, 150 mM sodium chloride, 1 mM DTT and protease inhibitors. After immunoprecipitation, the material was split to RNA and Protein extraction. Levels of OCT4 alternative transcripts were quantified by real time PCR in RNA sample pools extracted from samples immunoprecipitated with anti-LIN28A or IgG alone, without anti-LIN28A.

Polysome profile analysis

For polysome profile analysis cell extracts from Daoy cells was used. Following addition of cycloheximide (100 µg/mL) to the cultures, cells were harvested and suspended in breaking buffer A (20 mM Tris/HCl pH 7.4, 50 mM NaCl, 10 mM $MgCl_2$, 1 mM DTT, 200 µg/mL heparin, 100 µg/mL cycloheximide, 1 mM PMSF) and lysed. Polysomes were separated by centrifugation at 190,000xg for 3 hours at 4 °C with a Beckman SW41 rotor. Gradients were fractionated and monitored at 254 nm with an absorbance monitor (BioRad, Hercules, CA, USA). Analysis of free ribosome subunits was performed using breaking buffer B (20 mM Tris/HCl pH 7.4, 50 mM NaCl, 400 mM EDTA, 1 mM PMDF, 1 mM DTT, 200 µg/mL heparin, 100 µg/mL cycloheximide). Proteins from each fraction (500 µL) were precipitated with 15% trichloroacetic acid and analyzed by western blot with specific antibodies.

Supplementary Table 1: Genes located in aberrant chromosomal regions common to Daoy and USP-13-Med cells after OCT4A overexpression

Gene	Cytoband	Condition in Daoy	Condition in USP-13-Med
**FAF1**	1p32.3	Deleted	Amplified
**CDKN2C**	1p32.3	Deleted	Amplified
**MIR548H2**	9p21.3	Amplified	Amplified
**IFNB1**	9p21.3	Amplified	Amplified
**IFNW1**	9p21.3	Amplified	Amplified
**IFNA21**	9p21.3	Amplified	Amplified
**IFNA4**	9p21.3	Amplified	Amplified
**IFNA7**	9p21.3	Amplified	Amplified
**IFNA10**	9p21.3	Amplified	Amplified
**IFNA16**	9p21.3	Amplified	Amplified
**IFNA17**	9p21.3	Amplified	Amplified
**IFNA14**	9p21.3	Amplified	Amplified
**IFNA5**	9p21.3	Amplified	Amplified
**KLHL9**	9p21.3	Amplified	Amplified
**IFNA6**	9p21.3	Amplified	Amplified
**IFNA13**	9p21.3	Amplified	Amplified
**IFNA2**	9p21.3	Amplified	Amplified
**IFNA8**	9p21.3	Amplified	Amplified
**IFNA1**	9p21.3	Amplified	Amplified
**LOC554202**	9p21.3	Amplified	Amplified
**IFNE**	9p21.3	Amplified	Amplified
**MIR31**	9p21.3	Amplified	Amplified
**MTAP**	9p21.3	Amplified	Amplified
**C9orf53**	9p21.3	Amplified	Amplified
**CDKN2A**	9p21.3	Amplified	Amplified
**CDKN2BAS**	9p21.3	Amplified	Amplified
**CDKN2B**	9p21.3	Amplified	Amplified
**DMRTA1**	9p21.3	Amplified	Amplified

Supplementary Table 2: Common differentially expressed genes in Daoy, D283Med and USP-13-Med cells after OCT4A overexpression. Gene expression profiling was carried out with three independent clones of each parental and respective OCT4A-overexpressing cells. See Supplementary_Table_2

Supplementary Table 3: Amount of differentially expressed genes and of genes located in chromosomal regions showing aberrant copy number, after OCT4A overexpression in medulloblastoma cells

Data	Daoy	USP-13-Med	D283Med
Amount of Differentially Expressed Genes (DEG)	852	710	736
Amount of Genes in Chromosomal Aberrations (SCA)	3063	40	0
Amount of Correlated DEG and genes in SCA	69	0	0

Supplementary Table 4: Differentially expressed genes located in regions with chromosomal aberrations in Daoy medulloblastoma cells overexpressing OCT4A

Gene	Expression (Fold-Change)	Mutation
C8orf31	2,02	Amplification
TRIM35	2,02	Amplification
EMG1	2,04	Amplification
CLU	2,08	Amplification
SLC25A37	2,08	Amplification
PSD3	2,09	Amplification
EXOSC4	2,11	Amplification
MRPS25	2,24	Amplification
SH2D4A	2,28	Amplification
MFSD3	2,36	Amplification
FBXL6	2,39	Amplification
SCARNA12	2,55	Amplification
TUBA3C	3,54	Amplification
CST3	4,73	Amplification
SLC25A24	–2,03	Deletion
PTPRA	–2,04	Deletion
PHTF1	–2,05	Deletion
MRAP2	–2,06	Deletion
NCOA1	–2,06	Deletion
LCA5	–2,07	Deletion
MAGI3	–2,08	Deletion
DENND2C	–2,1	Deletion
FAM102B	–2,1	Deletion
MID1	–2,1	Deletion
PIGK	–2,11	Deletion
PCNA	–2,13	Deletion
SORT1	–2,13	Deletion
PTGFRN	–2,14	Deletion
MANEA	–2,15	Deletion
PRDM2	–2,15	Deletion
ITGB1BP1	–2,16	Deletion
CRIM1	–2,18	Deletion
C4orf19	–2,23	Deletion
WDR47	–2,23	Deletion
PLEKHH2	–2,24	Deletion
PAX7	–2,27	Deletion
C20orf96	–2,3	Deletion
BMP2	–2,32	Deletion
ELOVL4	–2,34	Deletion
CD38	–2,36	Deletion
C20orf194	–2,37	Deletion
WBSCR17	–2,37	Deletion
RASSF2	–2,48	Deletion
ASAP2	–2,49	Deletion
TP73	–2,51	Deletion
ASXL2	–2,54	Deletion
MBOAT2	–2,61	Deletion
TBC1D19	–2,68	Deletion
VIPR1	2,69	Deletion
KIDINS220	–2,71	Deletion
WNT2B	–2,71	Deletion
NBPF4	–2,79	Deletion
TNS3	–2,8	Deletion
ADAM17	–2,91	Deletion
STK17A	–3,04	Deletion
CD101	–3,07	Deletion
XYLT1	–3,07	Deletion
AK5	–3,11	Deletion
IAH1	–3,11	Deletion
SOD3	–3,15	Deletion
CPSF3	–3,18	Deletion
CSF1	–3,43	Deletion
TRIB2	–3,48	Deletion
CYTL1	–3,63	Deletion
IFI44L	–3,68	Deletion
LEPREL1	–3,72	Deletion
C7orf69	–4,59	Deletion
MXRA5	–7,96	Deletion
PROM1	–7,99	Deletion

*Genes differentially expressed by a ratio greater than 2.0 fold were compared with genes located in altered chromosome regions. Correlation was considered when amplifications were matched with increased gene expression or deletions were matched with decreased gene expression.

Supplementary Table 5: Primer sequences used in this study

Transcript	Primer Sequence
OCT4A-Forward	5′-TCGCAAGCCCTCATTTCACCA-3′
OCT4A-Reverse	5′-GGACTCCTCCGGGTTTTGCT-3′
OCT4B-Forward	5′-GCACTTCTACAGACTATTCCTTG-3′
OCT4B-Reverse	5′-AATACCTTCCCAAATAGAACCC-3′
OCT4B1-Forward	5′-AAATCCAGTCCCAGGACATCA-3′
OCT4B1-Reverse	5′-CTGAATAACTTCCCTGGGGG-3′
LIN28A-Forward	5′-CCAGTGGATGTCTTTGTGCACC-3′
LIN28A-Reverse	5′-GTGACACGGATGGATTCCAGAC-3′
β-ACTIN-Forward	5′-CGACAGGATGCAGAAGGAG-3′
β-ACTIN-Reverse	5′-TCCTGCTTGCTGATCCACAT-3′

Supplementary Data 1: Copy number aberrations in Daoy and USP-13-Med cells detected after OCT4A overexpression. See Supplementary_Data_1

Supplementary Video 1: Time lapse of tumorsphere formation *in vitro*. Photomicrographs were taken every 30 minutes for 4 days. The video shows that OCT4A overexpressing cells generated more tumorspheres than control cells. Increased tumorsphere size results from both enhanced self-renewal of tumor cells and fusion of neighboring spheres. See Supplementary_Video_1

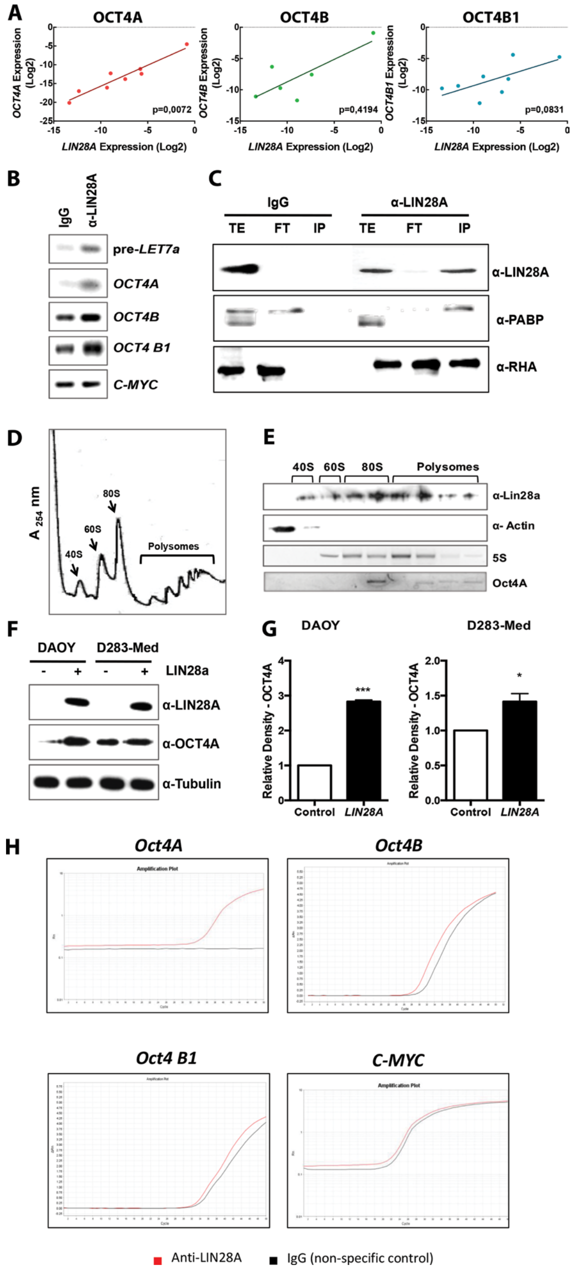

Supplementary Figure 1: LIN28A regulates OCT4A isoform post-transcriptionally and enhances OCT4A translation in medulloblastoma. (**A**) Significant positive correlation between LIN28A and OCT4A expression in medulloblastoma specimens. (**B**) LIN28A specifically binds OCT4A transcripts. LIN28 was immunoprecipitated (IP) from Daoy cell extracts and the corresponding bound RNA was extracted and analyzed by qRT-PCR. Pre-Let-7a and C-MYC transcripts were respectively used as positive or negative controls of specific LIN28A RNA binding. (**C**) LIN28A interacts with PABP – Poly(A) Binding Protein and RHA – RNA Helicase A. Protein detection by Western Blot. TE-Total Extract from Daoy cells, FT-Flow Through fraction and IP-Immunoprecipitated Fraction. (**D**) Polysomal profiling of Daoy cells. Detection of LIN28A cosedimenting with 40 S, 60 S, 80 S ribosomal particles and polysomal fractions by Western Blot. Actin was used as a control of protein not involved in translation. (**E**) Detection of Oct4A transcripts cosedimenting with LIN28A within 80S and polysomal fractions of polysomal gradients. (**F**) Western Blot of cell extracts from Daoy and D283Med cells overexpressing LIN28A reveal a positive upregulation of OCT4A protein levels by LIN28A in medulloblastoma. (**G**) Densitometry of Western Blot protein bands showing increased levels of OCT4A upon LIN28A overexpression in Daoy and D283Med cells. The bars represent mean ± SEM of three independent experiments. *$p < 0.05$, ***$p < 0.001$ (**H**) LIN28 was immunoprecipitated (IP) from Daoy cell extracts and the corresponding bound RNA was extracted and analyzed by qRT-PCR. Amplification plots of OCT4A, OCTB and OCTB1 are shown. C-MYC transcripts were also evaluated as negative control of specific LIN28A RNA binding. Red curves represent amplification from RNA bound to LIN28A; black curves represent amplification from RNA bound to IgG only, without anti-LIN28A.

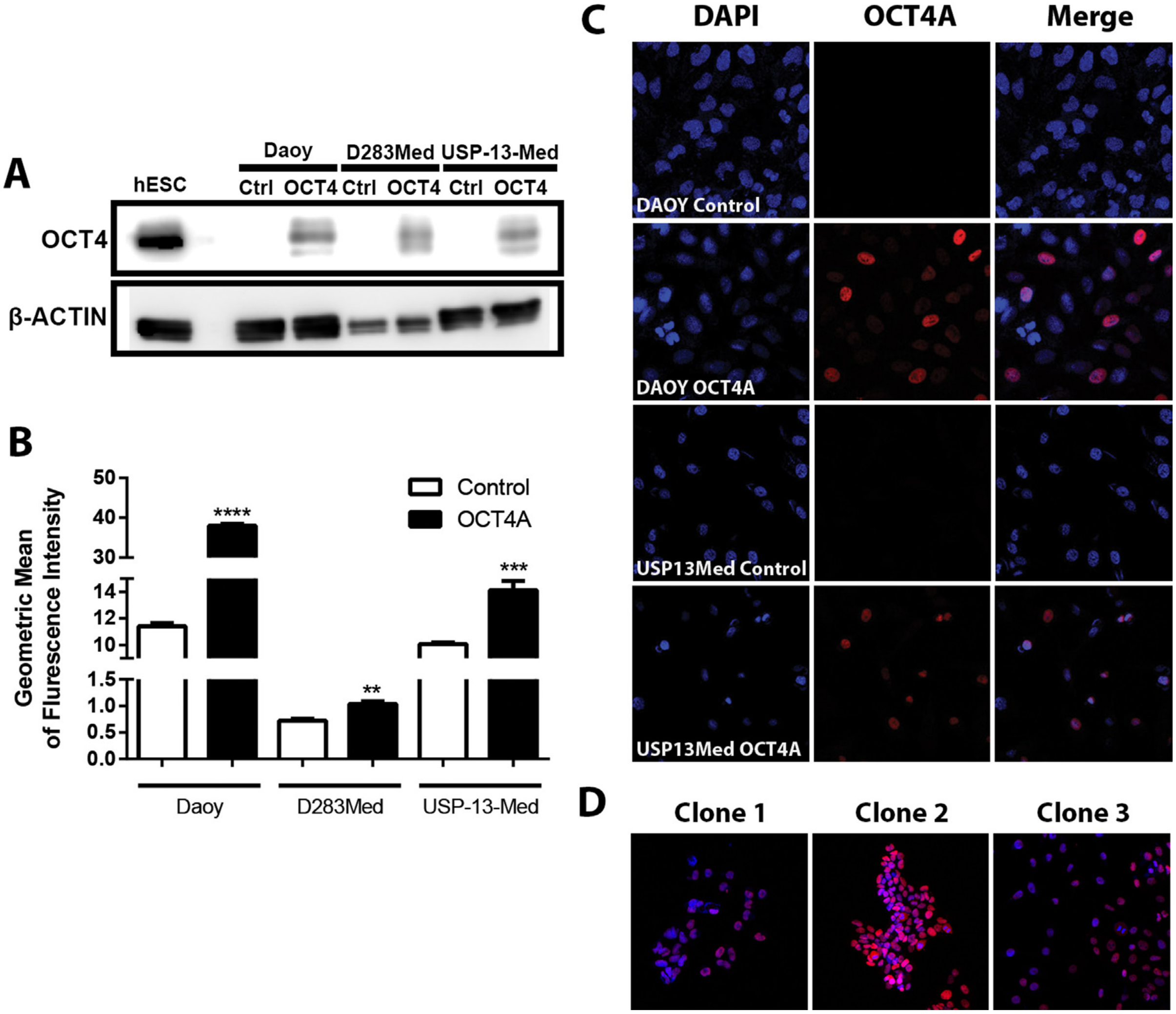

Supplementary Figure 2: OCT4A overexpression in medulloblastoma cell lines. (**A**) OCT4A protein levels in medulloblastoma cells and human embryonic stem cell (hESC), assessed by western blotting. Ctrl: Control; OCT4A: OCT4A Overexpression. (**B**) Geometric mean of fluorescence intensity obtained by flow cytometry in control and OCT4A overexpressing cells. (**C**) Immunofluorescence detection of nuclear OCT4A in Daoy and USP-13-Med cells overexpressing OCT4A. DNA was stained with DAPI (Blue). Cells were incubated with antibody against OCT4A (Red). (**D**) Immunofluorescence detection of homogenous clone-derived cell lines from Daoy OCT4AHi. DNA was stained with DAPI (Blue). Cells were incubated with antibody against OCT4A (Red).

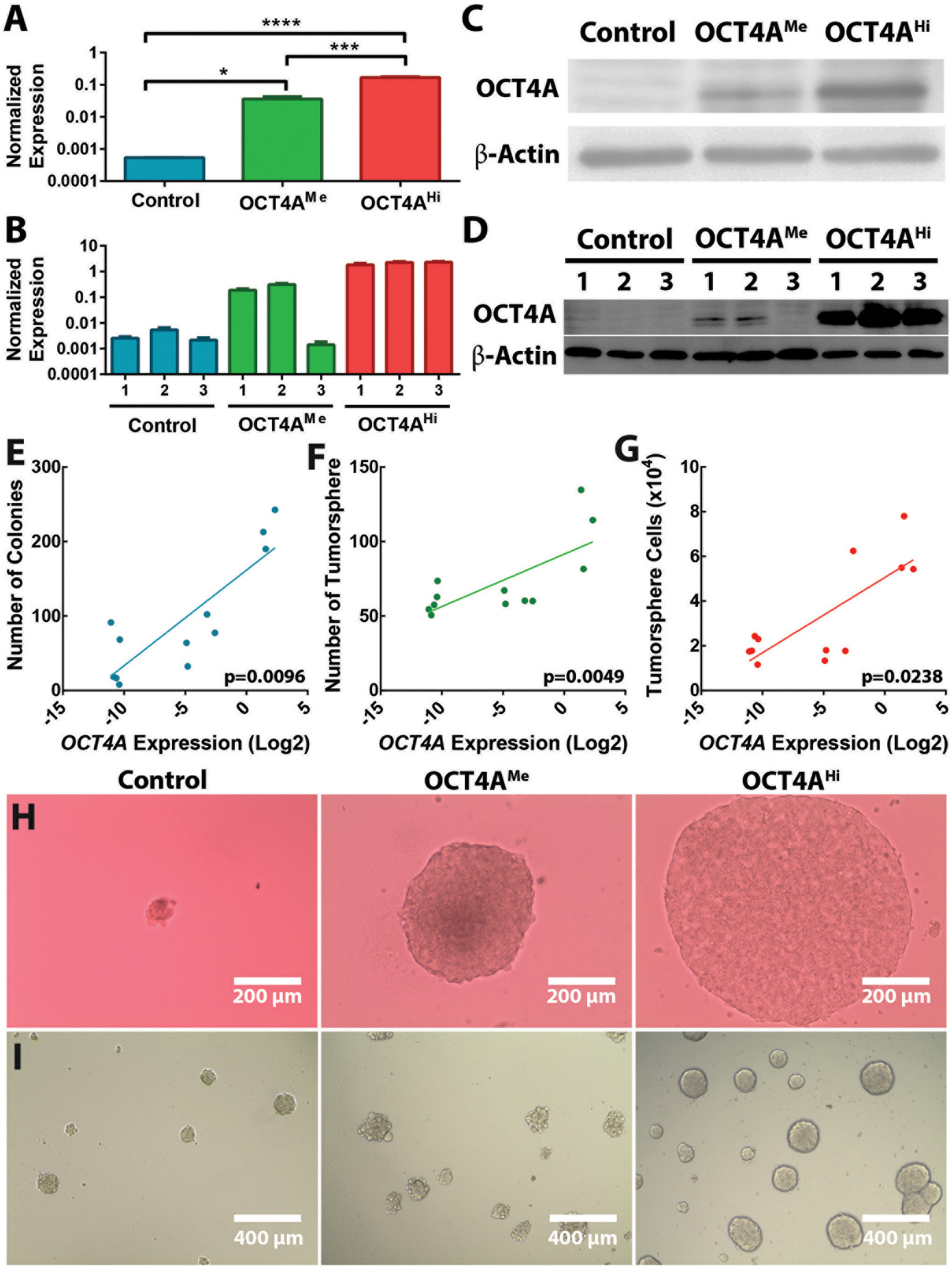

Supplementary Figure 3: OCT4A affects colony and tumorsphere formation in an expression level-dependent fashion. (**A**) Normalized OCT4A expression in parental Daoy cells displaying increasing levels of OCT4A overexpression (OCT4AMe and OCT4AHi) relative to Control Cells; and (**B**) in three distinct clonal cell lines derived from each of these groups (Control, OCT4AMe and OCT4AHi). (**C**) Respective OCT4A protein levels in parental and in (**D**) clonal cell lines, as detected by Western Blot. Positive correlation between OCT4A expression and (**E**) colony formation capability, (**F**) tumorsphere capability, (**G**) total amount of tumorsphere cells. (**H**) Colony size and (**I**) tumorsphere size were also affected by OCT4A in an expression level-dependent manner. $*p < 0.05$, $***p < 0.001$.

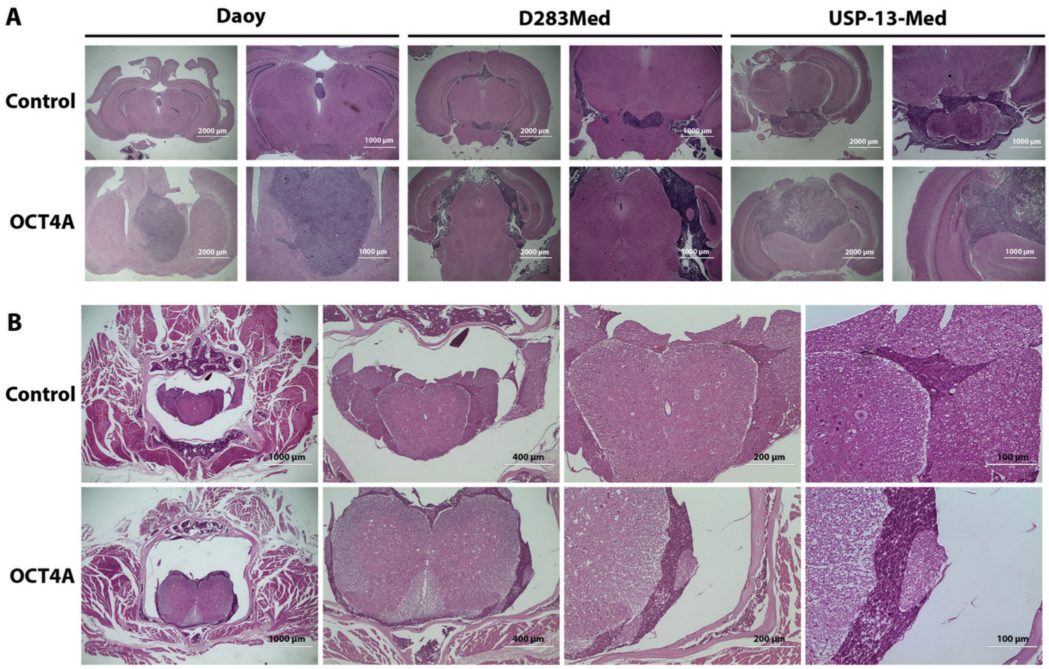

Supplementary Figure 4: Histological analysis of orthotopic tumors derived from human medulloblastoma cells. (**A**) Representatives images of brain tumors in mice. (**B**) Detection of metastatic tumors in spinal cord of animals orthotopically injected with D283Med cells.

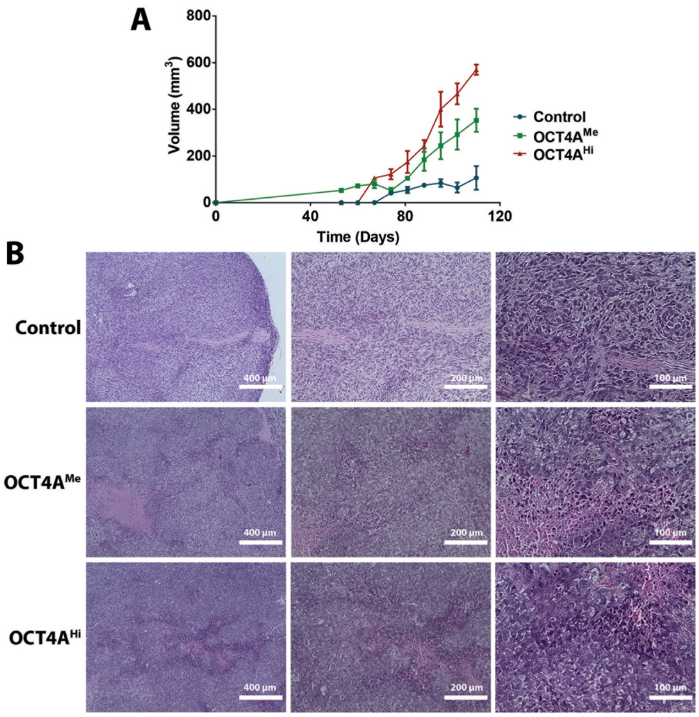

Supplementary Figure 5: Expression level-dependent effect of OCT4A in tumorigenesis. (**A**) Positive correlation between OCT4A expression and tumorigenesis. Tumor volume was measured weekly during 110 days after subcutaneous injection of medulloblastoma cells in Balb/c nude mice. Control and OCT4AHi curves were previously presented in Figure 4. (**B**) Representative images of subcutaneous tumors after HE staining. Tumors derived from OCT4A overexpressing cells displayed a more heterogeneous phenotype, increased inflammatory and hemorrhagic areas, compared with tumors from control tumor cells.

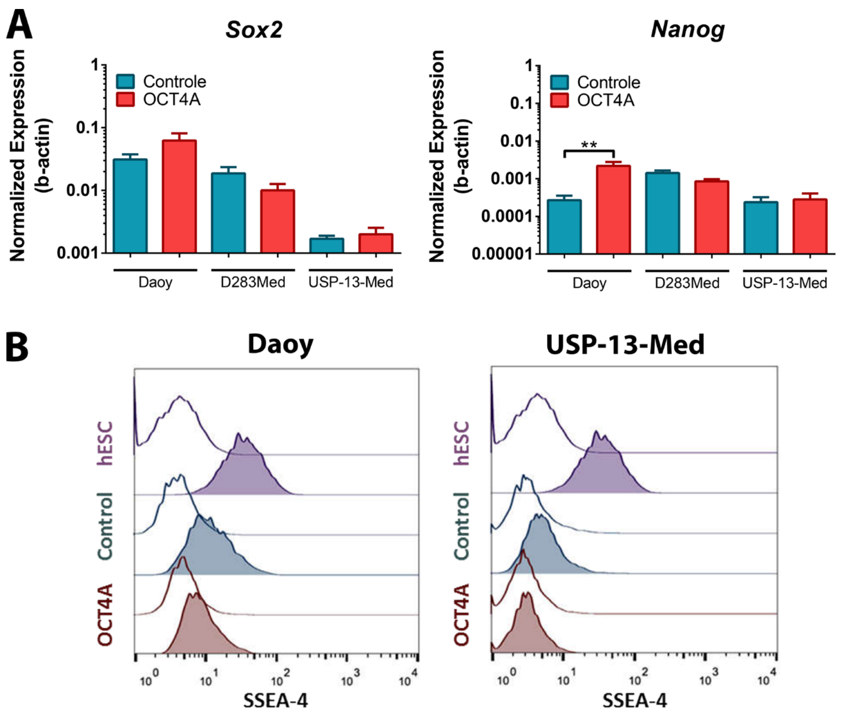

Supplementary Figure 6: Expression of pluripotency markers in OCT4A-overexpressing medulloblastoma cells. (**A**) Normalized Sox2 and Nanog expression in human medulloblastoma cells show no consistent upregulation due OCT4A increased levels $**p < 0.01$. (**B**) Histogram distribution of fluorescence intensity obtained by flow cytometry analysis of control and OCT4A-overexpressing cells labeled with anti-SSEA-4. hESC was used as positive control. Open histogram: Negative control. Filled histogram: Experimental condition.

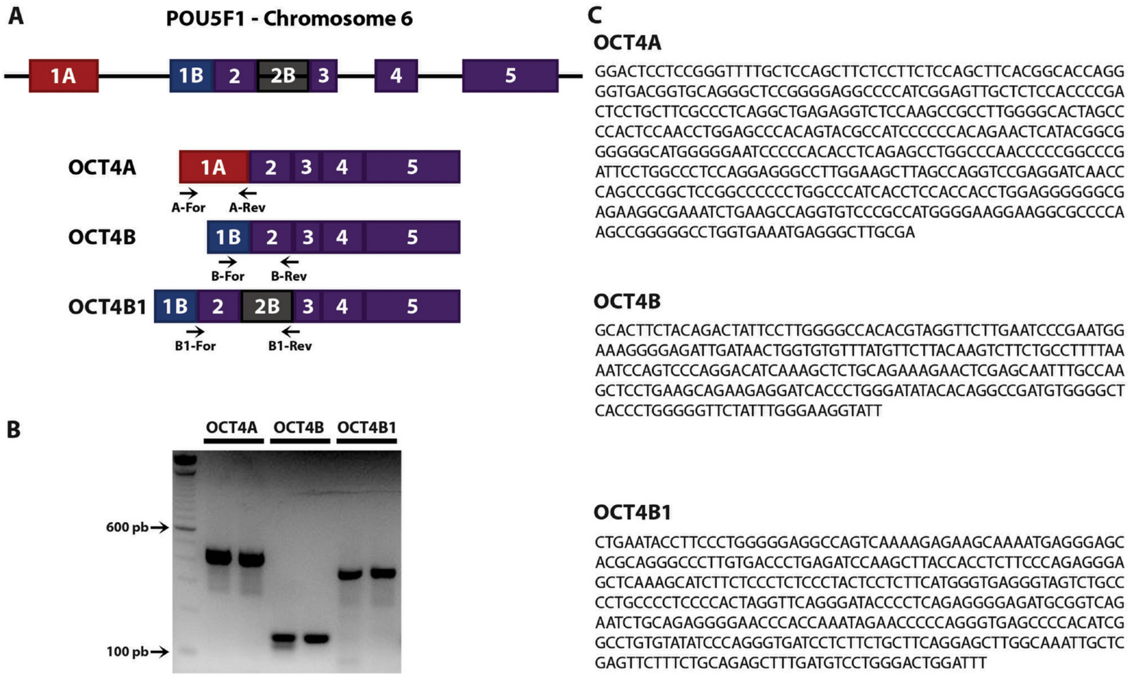

Supplementary Figure 7: Primer design and confirmation of amplification specificity of OCT4 transcript variants. (**A**) Location of primers in OCT4 transcripts. (**B**) Amplicons derived from OCT4 transcript amplification qRT-PCR reactions (Oct4A: 459 pb; Oct4B: 139 pb; Oct4B1: 375 pb). (**C**) Sanger DNA sequencing of amplicons confirming OCT4 transcript identities.
